# Novel Predictors of Future Vascular Events in Post-stroke Patients—A Pilot Study

**DOI:** 10.3389/fneur.2021.666994

**Published:** 2021-06-18

**Authors:** Diana Schrick, Erzsebet Ezer, Margit Tokes-Fuzesi, Laszlo Szapary, Tihamer Molnar

**Affiliations:** ^1^Department of Anesthesiology and Intensive Care, Medical School, University of Pécs, Pécs, Hungary; ^2^Department of Laboratory Medicine, Medical School, University of Pécs, Pécs, Hungary; ^3^Department of Neurology, Medical School, University of Pécs, Pécs, Hungary

**Keywords:** recurrent stroke, vascular event, platelet function, platelet reactivity, outcome

## Abstract

**Introduction:** A modified platelet function test (mPFT) was recently found to be superior compared to impedance aggregometry for selection of post-stroke patients with high on-treatment platelet reactivity (HTPR). We aimed to explore some peripheral blood cell characteristics as predictors of recurrent ischemic episodes. The predictive value of mPFT was also assessed in a cohort followed up to 36 months regarding recurrent ischemic vascular events.

**Methods:** As a novelty, not only whole blood (WB), but after 1-h gravity sedimentation the separated upper (UB) and lower half blood (LB) samples were analyzed including neutrophil antisedimentation rate (NAR) in 52 post-stroke patients taking clopidogrel. Area under the curve (AUC, AUC_upper_ and AUC_lower_, respectively) was separately measured by Multiplate in the WB, UB and LB samples to characterize *ex vivo* platelet aggregation in the presence of ADP. Next, the occurrence of vascular events (stroke, acute coronary syndrome, ACS) were evaluated during 36-month follow-up.

**Results:** A total of 11 vascular events (stroke *n* = 5, ACS *n* = 6) occurred during the follow-up period. The AUC_upper_ was significantly higher in patients with recurrent stroke compared to those with uneventful follow-up (*p* = 0.03). The AUC_upper_ with a cut-off value ≥70 based on the mPFT, was able to predict all stroke events (*p* = 0.01), while the total vascular events were independently predicted by NAR with a sensitivity of 82% and specificity of 88%.

**Conclusions:** A combination of NAR reflecting the inflammatory state and AUC_upper_ indicating HTPR may provide a better prediction of recurrent ischemic events suggesting a better selection of patients at risk, thus providing an individually tailored vascular therapy.

## Introduction

Despite successful recanalization strategies either with thrombolysis or using endovascular treatments for acute ischemic stroke, the eventual outcome of patients is far from desirable (1). Among many factors, some peripheral blood cells may play a pivotal role in post-procedural microcirculatory alterations contributing to the outcome (2, 3). A higher incidence of recurrent cerebral ischemia was described in post-stroke patients with high on-treatment platelet reactivity (HTPR) (4, 5). Numerous tests assessing *ex vivo* platelet reactivity were used for identification of patients at risk for HTPR (6). However, the prevalence of HTPR was shown to vary depending on the definition and assay used (7). A modified platelet function test (mPFT) was recently found to be superior compared to conventional Multiplate Electrode Aggregometry for selection of post-stroke patients with HTPR (8).

Therefore, we aimed to explore some peripheral blood cell characteristics including platelets and neutrophils as predictors of recurrent ischemic episodes and factors contributing to the outcome. The predictive value of the mPFT as a point-of-care test (POCT) was also compared to conventional Multiplate Electrode Aggregometry in a cohort followed up to 36 months regarding recurrent ischemic vascular events.

## Materials and Methods

### Subjects

The study protocoll was approved by the University of Pecs Clinical Centre Regional and Institutional Research Ethics Comittee (8). Written informed consent was obtained from each patient. A total of 52 patients (age: 66 ± 8 years, male: 31) on antiplatelet therapy (75 mg clopidogrel once daily) due to secondary stroke prevention were prospectively recruited into this study. The selected patients with previous anterior circulation large artery atherothrombosis were on regular medical check-up at the Outpatient Clinic of the Department of Neurology. Fasting venous blood samples were taken *via* a 21G peripheral venous canula from each patient and healthy subjects. Patients were instructed to take their daily clopidogrel at least 2 h prior to blood sampling. Exclusion criteria were acute infection and acute vascular events, such as acute ischemic stroke (AIS), transient ischemic attack (TIA), acute myocardial infarction (AMI), acute coronary syndrome (ACS), thrombocytopenia (platelet count <150G/l), congenital platelet abnormalities, congenital disorder of haemostatis (e.g., hemophilia), anemia and patients on medical therapy influencing blood coagulation (e.g., oral anticoagulants, novel oral anticoagulants, non-steroid antiinflammatory drugs). The comorbidities, medications and smoking status were also recorded. Besides, the baseline erythrocyte sedimentation rate (ESR), C-reactive protein (CRP), and total blood count were measured. Next, the incidence of vascular events (ACS and recurrent ischemic stroke) in the total study population was evaluated in a 36-month follow-up. ACS was defined by using the ACC/AHA guidelines (shortly: based on clinical history, ECG results, levels of cardiac markers, and the results of stress testing). Each recurrent ischemic stroke was confirmed by neuroimaging (CT or MRI). All patients with either ACS or recurrent ischemic stroke were presented at the Emergency Department and underwent a careful clinical evaluation then archived by an electronic database.

### Blood Sampling

Venopuncture was performed from the cubital vein after short time strangulation of the arm with 21G BD vacutainer needle. The total blood count was measured after taking into vacutainers with EDTA (REF: 368856, 5.4md EDTA). Whole blood for platelet aggregometry was also taken into hirudin containing tube for Multiplate Electrode Aggregometry.

### Platelet Antisedimentation Rate, Neutrophil Antisedimentation Rate

Modified whole blood gravity sedimentation technique was developed for studying platelet and neutrophil sedimentation properties (8). After 1-h gravity sedimentation, the upper and lower half of the venous blood column was separately removed from the EDTA sedimentation tube and transferred to another EDTA tube for further analysis. An automatic cell counter system (Sysmex XN 9000, Sysmex Co, Japan, 2017) was applied to measure the upward floating (ascending) and sinking (non-ascending) cells in the separated samples. Next, the platelet antisedimentation rate (PAR, %), leukocyte antisedimentation rate (LAR,%) and neutrophil antisedimentation rate (NAR, %) were, respectively, calculated based on the equation:

$$\begin{array}{l} \frac{\text{cell coun}{\text{t}}_{\text{upper}}-\text{cell coun}{\text{t}}_{\text{lower}}}{\text{cell coun}{\text{t}}_{\text{upper}}+\text{cell coun}{\text{t}}_{\text{lower}}}\text{ X}100 \end{array}$$

### Multiplate Electrode Aggregometry

Platelet function test in the whole blood was perfomed from a hirudin containing tube with a Multiplate® Analyzer (Roche Diagnostics, Mannheim, Germany). Another hirudin containing tube was used for sedimentation, similarly to whole blood sedimentation in the EDTA-tube. After 1-h gravity sedimentation, the blood coloumn was devided into upper and lower samples. Platelet aggregometry was uniformly performed 60 min after blood sampling using adenosine diphosphate (ADP; 6.5 M) as agonist. As a novelty, not only whole blood, but after 1-h gravity sedimentation the separated upper and lower half blood samples were simultaneously analyzed in each post-stroke patient taking clopidogrel. Aggregation level was expressed as the area under the curve (AUC). AUC was calculated by a Multiplate® Analyzer using the product of aggregation unit (AU) × time (minutes) (9). After ADP stimulation, the normal aggregation range was expected as AUC: 53–122 according to the manufacturer (9). Based on the whole blood AUC, patients on clopidogrel were categorized as responder cases with AUC <53 and resistant cases representing HTPR with an AUC ≥53 (10).

### Statistical Analysis

Data were evaluated using SPSS software package (Version 19.0, SPSS Inc, Chicago, USA). Categorical data were summarized by means of absolute and relative frequencies (counts and percentages). Quantitative data were presented as median and 25th−75th percentiles, as well as mean ± SD. The Kolmogorov-Smirnov test was applied to check for normality. Chi-square test for categorical data and Student-*t* test for continuous data were used for analysis of demographic and clinical factors. Non-parametric Mann-Whitney *U* test was used for not normally distributed parameters. Correlation analysis was performed calculating Spearman's correlation coefficient (rho). A *p*-value <0.05 was considered statistically significant.

## Results

A total of 52 convalescent ischemic stroke patients were prospectively enrolled into this pilot study. All patients have been previously suffered from large vessel occlusion. The demography and clinical data of the study population is summarized in Table 1. A total of 11 vascular events (stroke n=5, ACS n=6) occured during 36-month follow-up. Of the antisedimentation rate indices, only NAR showed significant difference between “uneventful” vs. “vascular events” groups (Table 1). It is noteworthy that no difference was observed between the baseline blood count parameters (platelet, leukocyte, neutrophil), while a trend-like difference was observed in the ESR (Table 1). The AUC in the whole blood, and in the upper and lower samples after 1-h gravity sedimentation in the total population, and also a comparison between uneventful vs. stroke + ACS as well as uneventful vs. recurrent stroke alone subgroups are shown in (Table 2). The AUC_upper_ was significantly higher in patients with recurrent stroke compared to those with uneventful follow-up (*p* = 0.03) (Table 2).

**Table 1:** Demography and clinical data of the total population, and comparison between patients without vs. with recurrent vascular events during 36-month follow-up.

	**Total population** ***n* = 52**	**Uneventful** ***n* = 41**	**Vascular events** ***n* = 11**	***p*-value**
age	66 ± 8	66 ± 8	66 ± 9	0.937
male, *n*	34	26	8	0.564
hypertension, *n*	51	40	11	0.601
diabetes mellitus, *n*	14	10	4	0.427
smoking, *n*	11	9	2	1.000
ESR	12 (8–18)	10 (8–16)	18 (14–29)	0.063
CRP	1.9 (0.7–4.6)	1.8 (0.7–5.0)	2.2 (1.4–3.35)	0.614
PLT	224 (200–260)	224 (207–251)	243 (171–300)	0.805
PAR	67.9 (63.1–73.4)	67.8 (62.9–73.5)	70.0 (64.6–72.6)	0.614
WBC	6.8 (5.8–8.0)	6.6 (5.8–7.9)	7.4 (5.5–10.6)	0.420
LAR	35.7 (23.7–46.3)	36.2 (24.7–46.4)	34.4 (24.0–43.5)	0.806
neutrophil	61.8 (55.4–66.4)	62 (56–67)	58 (51–62)	0.317
NAR	−1.1 (−4.8–6.5)	0.9 (−3.9–7.2)	−5.2 [−6.8–(−4.7)]	0.001

*Vascular events, recurrent stroke, and de novo acute coronary event; ESR, erythrocyte sedimentation rate; CRP, C-reactive protein; PLT, platelet; PAR, platelet antisedimentation rate; WBC, white blood cell; LAR, leukocyte antisedimentation rate; NAR, neutrophil antisedimentation rate. Data are presented as median and 25th−75th percentiles, except age as mean ± SD*.

**Table 2:** Area under the curve (AUC) in the whole blood, and AUC in the upper and lower samples after 1-h gravity sedimentation in the total population and comparison between uneventful vs. stroke + ACS as well as uneventful vs. recurrent stroke subgroups.

	**Total population** ***n* = 52**	**Uneventful** ***n* = 41**	**Stroke + ACS** ***n* = 11**	***p*-value**
AUC	40.5 (27–53.5)	40 (27–54)	42 (32.5-44)	0.866
AUC_upper_	56 (22.5–76.5)	51.5 (19.5–77.5)	65 (42–75.5)	0.247
AUC_lower_	18 (13.5–22)	18 (14–23)	17 (13–20)	0.567
	**Total population** ***n*** **=** **52**	**Uneventful** ***n*** **=** **41**	**Stroke events** ***n*** **=** **5**	***p*****-value**
AUC	40.5 (27–53.5)	39 (27–53)	43 (42–44)	0.347
AUC_upper_	56 (22.5–76.5)	49 (21–74)	77 (71–92)	*0.020*
AUC_lower_	18 (13.5–22)	18 (14–22)	17 (11–19)	0.763

*AUC, area under the curve measured by Multiplate analyzer; AUC_upper_, AUC in the upper sample after 1-h gravity sedimentation; AUC_lower_, AUC in the lower sample after 1-h gravity sedimentation*.

### Independent Predictors

Based on ROC analysis, the AUC_upper_ with a cut-off value ≥70 measured by the mPFT was able to predict recurrent stroke events (*p* = 0.01) with the best sensitivity and specificity. Moreover, the total vascular events (stroke+ACS) was independently predicted by NAR with a sensitivity of 82% and specificity of 88% using a multiple regression analysis including relevant covariates (Table 3). Neither recurrent stroke nor ACS showed association with HTPR status defined by AUC>53 measured by the Multiplate in the whole blood.

**Table 3:** Predictors of vascular events during 36-month follow-up.

	****β****	***p*-value**	**OR**	**95% CI**	
age	−0.071	0.353	0.931	0.801	1.082
AUC	−0.046	0.320	0.955	0.871	1.046
AUC_upper_	−0.083	0.031	1.086	1.007	1.171
NAR	−0.489	0.032	0.613	0.392	0.960

*AUC, area under the curve measured by Multiplate analyzer; AUC_upper_, AUC in the upper sample after 1-h gravity sedimentation; NAR, neutrophil antisedimentation rate; OR, odds ratio, 95%CI, 95% confidence interval. Binary logistic regression analysis*.

### Cut-Off Values of Predictors

**Figure 1:**
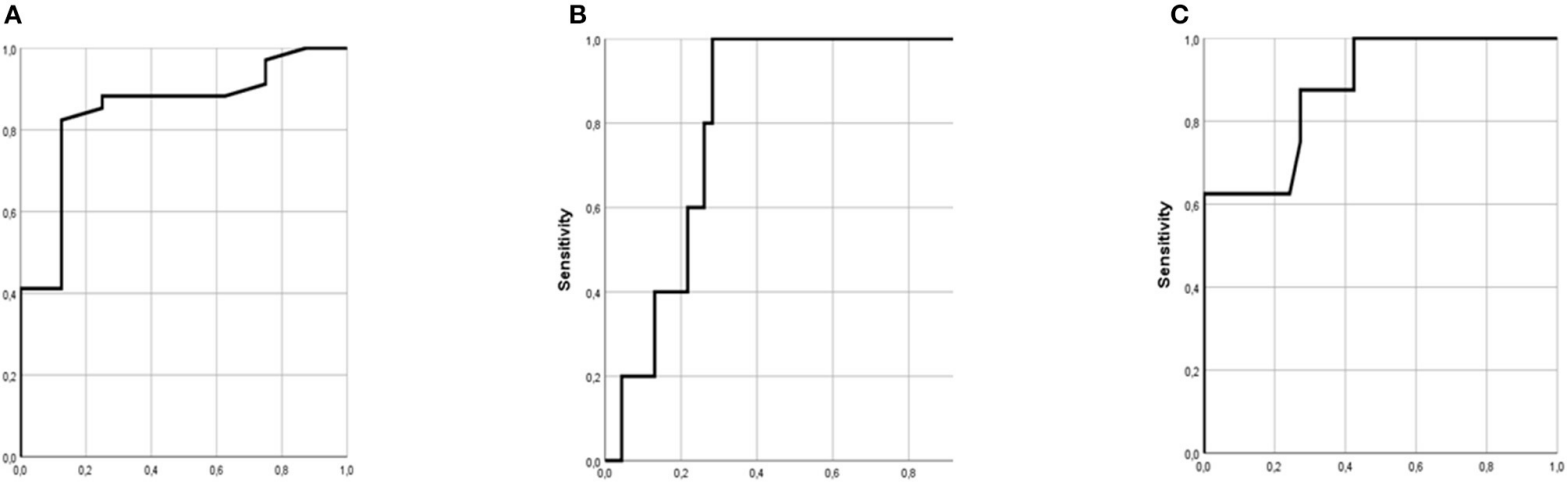
ROC curves of variables predicting recurrence of vascular events during follow-up. **(A)** ROC of neutrophil antisedimentation rate (NAR) (Area: 0.847, *p* = 0.002, 95%CI: 0.703–0.992). **(B)** ROC of platelet function test based on impedance aggregometry in the upper blood sample (AUC_upper_) after 1-h gravity sedimentation (Area: 0.813, *p* = 0.023, 95%CI: 0.689–0.937). **(C)** ROC of predicted probability of the combination of NAR and AUC_upper_ (Area: 0.881, *p* = 0.001, 95%CI: 0.754–1.0).

The ROC curves of variables predicting recurrence of vascular events during follow-up are shown in Figure 1. In this cohort, NAR with a cut-off ≥ −0.431 independently predicted recurrence of total vascular events (stroke + ACS, *n* = 11) with a sensitivity of 82% and specificity of 88% during 36-month follow-up (Area: 0.847, *p* = 0.002, 95%CI: 0.703–0.992) (Figure 1A). Furthermore, ROC of platelet function test based on impedance aggregometry in the upper blood sample after 1-h gravity sedimentation revealed, that AUC_upper_ with a cut-off ≥ 70 predicted recurrent stroke with a sensitivity of 80% and specificity of 74% during 36-month follow-up (Area:0.813, *p* = 0.023, 95%CI:0.689–0.937) (Figure 1B). Finally, a more precise model was created, when a ROC analysis was performed with predicted probablity of the combination of NAR and PFT_upper_ (Area:0.881, *p* = 0.001, 95%CI:0.754–1.0) (Figure 1C).

## Discussion

Activation of neutrophils reflected by NAR was shown here as the most sensitive marker of recurrence of ischemic cerebral episodes in post-stroke patients taking clopidogrel. Both, animal and clinical data support the pivotal role of activated peripheral blood cells (e.g., neutrophils, monocytes, platelets) in neuroinflammation due to ischemic stroke (2, 3, 11). One side, the dynamic microcirculatory stall phenomenon in the hyperacute stage can be a contributing factor to ongoing penumbral brain injury (2, 12), on the other side the sustained detrimental effects of activated leukocytes in the systemic circulation carries a constant risk in patients with chronic inflammatory state (e.g., vascular diseases) (13). Interestingly, a downward motion of neutrophils during 1-h gravity sedimentation expressed by a negative value of NAR was observed in those patients who suffered from composite vascular events during 36-month follow-up. In contrast, an upward motion of both, leukocytes and platelets proportionally to their activation was described previously in acute ischemic stroke (3), post-stroke infection (14) and burn patients (15). Neither LAR, nor PAR was found to be predictive for future vascular events in convalescent stroke patients suggesting that leukocytes and platelets exert their actions predominantly in the acute phase of stroke. Our finding also suggests that neutrophils are important markers of stroke outcome as their predictive role was recently shown in patients with acute coronary syndrome (16).

Numerous data highlight that a high proportion of patients with cardiovascular diseases have *ex vivo* HTPR on their prescribed antiplatelet regimen (4, 5, 7). Although several studies show an increased rate of recurrent cerebrovascular ischemic events in patients presenting HTPR, the diagnostics of HTPR has been unsolved so far (4, 17). Here, the state of clopidogrel resistance based on Multiplate electrode aggregometry from the whole blood was not able to predict recurrent stroke. However, a higher AUC (≥70 as a cut-off value) from the separated upper blood sample after 1-h gravity sedimentation emerged as a novel independent predictor of future stroke episode in our study. This observation suggests that the upward motion of platelets might be associated with increased thrombotic tendency. Further studies are needed to explore the characteristics of this subpopulation of platelets and their impact on post-stroke complications and outcome. When the combination of NAR and PFT_upper_ was used in the statistical model, the predicted probability of a future vascular event was even more accurate.

In summary, while AUC_upper_ indicates more precise definition of HTPR, NAR rather reflects the inflammatory state in post-stroke patients (18). Based on this small, single-center pilot study, these novel markers may provide a better prediction of recurrent ischemic events leading to a better selection of patients at risk and providing an individually tailored vascular therapy including antiplatelet and anti-inflammatory regimens (17, 19).

## Limitations

This is a small prospective cohort with a 36-month follow-up focusing primarily on recurrent coronary and cerebral ischemic episodes which required hospitalization. However, the silent ischemic lesion recurrence on MRI was not explored here. Therefore, a large, adequately sized, prospective multicenter study is needed to determine whether these novel assessments of HTPR in conjunction with pharmacogenetic and neuroimaging (diffusion weighted imaging, DWI) data, improves our ability to predict the risk of recurrent vascular events in patients with cardiovascular diseases. Although the interaction between inflammation and ischemic stroke is multifaceted, a better understanding of such mechanisms may lead to enhanced secondary prevention including immunomodulatory approaches and more precise antiplatelet therapy (20, 21).

## Data Availability Statement

The raw data supporting the conclusions of this article will be made available by the authors, without undue reservation.

## Ethics Statement

The studies involving human participants were reviewed and approved by Local Ethics Committee of the University of Pécs. The patients/participants provided their written informed consent to participate in this study.

## Author Contributions

DS, EE, LS, and TM conceived, designed and coordinated the study, participated in acquisition, and interpretation of data. DS and TM drafted the manuscript. MT-F performed the laboratory measurements. MT-F and TM participated in the statistical analysis. All authors read and approved the manuscript.

## Conflict of Interest

The authors declare that the research was conducted in the absence of any commercial or financial relationships that could be construed as a potential conflict of interest.
